# Facelift as a novel surgical treatment for HIV-associated facial lipodystrophy: a case report and literature review

**DOI:** 10.1093/jscr/rjaf683

**Published:** 2025-09-02

**Authors:** Daniyal Ashraf, Rushabh Shah, Taj Tomouk, Richard Price

**Affiliations:** School of Clinical Medicine, University of Cambridge, Cambridge CB2 0SP, Cambridgeshire, United Kingdom; Department of Plastic and Reconstructive Surgery, Cambridge University Hospitals NHS Foundation Trust, Cambridge CB2 0QQ, Cambridgeshire, United Kingdom; Department of Plastic and Reconstructive Surgery, Cambridge University Hospitals NHS Foundation Trust, Cambridge CB2 0QQ, Cambridgeshire, United Kingdom; Department of Plastic and Reconstructive Surgery, Cambridge University Hospitals NHS Foundation Trust, Cambridge CB2 0QQ, Cambridgeshire, United Kingdom

**Keywords:** HIV-associated lipodystrophy, facelift, antiretroviral therapy, Frey’s syndrome, facial rejuvenation

## Abstract

Human immunodeficiency virus (HIV)-associated lipodystrophy is a well-recognized complication of long-term antiretroviral therapy (ART), characterized by abnormal fat distribution. In the head and neck, it typically present as an obtuse heavy neck combined with accelerated midfacial atrophy, causing significant aesthetic concerns and psychosocial distress. Conventional treatments, including ART modifications, have limited effectiveness, and changes are often irreversible. We present a novel surgical approach, a modified facelift, to manage facial lipodystrophy in a 57-year-old HIV-positive man. Following unsuccessful non-surgical interventions, the facelift provided substantial aesthetic improvements and addressed functional concerns associated with coincidental Frey’s syndrome. Postoperative recovery was uneventful, and at 6-month follow-up, the patient reported sustained aesthetic satisfaction and significant symptomatic improvement. This case demonstrates that a modified facelift is a promising low-morbidity surgical option for addressing complex facial lipodystrophy in HIV patients, offering durable cosmetic and functional outcomes where traditional methods have failed.

## Introduction

Human immunodeficiency virus (HIV)-associated lipodystrophy is a well-recognized complication of long-term antiretroviral therapy (ART), typically presenting as fat loss (lipoatrophy), fat accumulation (lipohypertrophy), or a combination of both [1]. Whilst the advent of newer ART regimens has reduced the incidence of lipodystrophy, the condition remains clinically significant, particularly in patients treated with older drug combinations [2]. In the head and neck region, lipodystrophy often manifests as midfacial hollowing and soft tissue ptosis alongside prominent cervical or parotid fullness [3]. These visible facial changes can be stigmatizing and are associated with depression, reduced self-esteem, and diminished adherence to ART [4].

Management remains challenging due to the multifactorial and incompletely understood aetiology of HIV-associated lipodystrophy [5]. Initial treatment focuses on modifying ART and implementing lifestyle changes, which may offer metabolic improvement or partial reversal of lipoatrophy but rarely address established disfigurement [6]. Lipohypertrophy, in particular, tends to persist despite ART adjustment [7].

When non-operative strategies fail, several interventions have been explored—including fillers, botulinum toxin, liposuction, and lipectomy—but each carries limitations. Fillers offer temporary improvement, whilst facial liposuction is associated with risks such as contour irregularity, overcorrection, and neurovascular injury [8].

In this report, we describe the use of a modified facelift procedure in a patient with ART-induced facial lipodystrophy and concurrent Frey’s syndrome. This low-morbidity surgical approach provided both aesthetic and functional benefit, in addition to addressing our patient’s co-incidental Frey’s syndrome. To our knowledge, this is the first reported use of a facelift specifically for HIV-associated facial lipodystrophy with dual functional and cosmetic objectives.

## Case report

A man in his 50s was referred to our facial reconstruction clinic with facial lipodystrophy secondary to HIV, along with coexisting Frey’s syndrome. He had been diagnosed HIV-positive in 1992 and subsequently developed non-Hodgkin’s lymphoma, prompting initiation of ART after his CD4 count dropped to 10 cells/μL. At presentation, he had been on ART for 23 years, with well-controlled HIV and lymphoma in remission.

**Figure 1:**
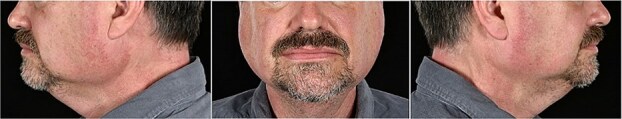
Photographs of frontal and side views of the patient before surgery.

Examination revealed bilateral soft fatty deposits in the parotid region, midfacial atrophy, and masseteric hypertrophy (Fig. 1). There was no cervical lymphadenopathy. Botulinum toxin was trialled to the masseters and parotids with minimal effect. Facial liposuction was deemed unsuitable due to risks of irregularity and neurovascular injury. A modified facelift was selected to address both the lipodystrophy and gustatory sweating by disrupting sympathetic branches.

Ultrasound demonstrated diffusely fatty parotid infiltration with normal overlying subcutaneous fat (5 mm bilaterally), confirming the distribution of lipodystrophic changes.

Under general anaesthetic, 40 ml of 0.25% bupivacaine with 1:200 000 adrenaline was infiltrated to the submental and subauricular areas. Liposuction was performed via submental stab incisions using a 3.7 mm cannula. A modified facelift was then performed via standard temporal, pre-auricular and post-auricular incisions. Subcutaneous dissection was carried out superficial to the platysma and SMAS. The SMAS overlying the parotids was elevated, excised, and re-sutured with Monocryl™. Redundant skin was excised as required, the earlobes re-inset, and bilateral drains placed.

**Figure 2:**
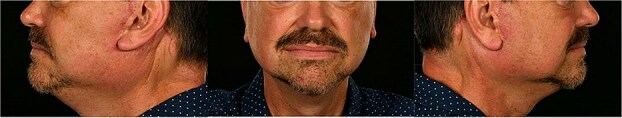
Six weeks post-operative photographs of the patient.

**Figure 3:**
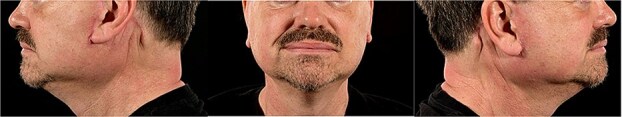
Six months post-operative photographs of the patient.

The patient was discharged the following day. At day 5, swelling was present but expected. By week 6, the scars had healed well and swelling had resolved (Fig. 2). At 6 months, the patient reported high satisfaction with aesthetic results and near-complete resolution of Frey’s syndrome symptoms (Fig. 3). Minor standing cones at the post-auricular scar were scheduled for revision under local anaesthetic.

## Discussion

ART, first introduced in the 1980s, transformed HIV from a terminal illness into a chronic, manageable condition. However, as with many long-term therapies, ART carries adverse effects—most notably metabolic changes and abnormal fat distribution. These may present as hyperlipidaemia, insulin resistance, and lipodystrophy, the latter formally recognized in 1998 [9].

Lipodystrophy may be lipoatrophic, lipohypertrophic, or mixed, with facial manifestations including midfacial hollowing, jowl ptosis, and an obtuse neck profile [10]. Such changes are not merely aesthetic: they are stigmatizing and psychologically distressing, significantly affecting quality of life and ART adherence [5]. Prevalence estimates vary widely (11%–83%) due to inconsistent diagnostic criteria [11].

The underlying mechanisms remain incompletely understood. Proposed contributors include HIV-induced inflammation, direct effects of viral proteins, mitochondrial toxicity from reverse transcriptase inhibitors, and genetic susceptibility [12]. Thymidine analogue NRTIs and protease inhibitors are particularly implicated [12]. Although newer regimens carry a lower risk, modifying ART may offer only modest improvements in established lipodystrophy [12].

Non-surgical approaches—diet, exercise, smoking cessation, and, in some countries, tesamorelin—can improve metabolic parameters but have limited cosmetic impact. In the UK, tesamorelin is not approved due to concerns about risk–benefit balance [13].

Surgical intervention is often necessary for correcting established deformity. Whilst liposuction and lipectomy are effective for large, discrete fat pads, they are poorly suited to the face due to the thin skin envelope, diffuse fat distribution, and proximity to critical neurovascular structures [10]. Filler injections, though popular, offer only temporary correction and require ongoing maintenance [14]. Autologous fat grafting has gained attention but introduces risks of fat resorption, variable outcomes, and undesirable fullness in cases of existing lipohypertrophy [15].

In this case, we employed a modified cervicofacial facelift with limited neck liposuction to manage facial lipodystrophy in an HIV-positive man. This offered effective redraping of soft tissues, aesthetic rejuvenation, and resolution of coincidental Frey’s syndrome. By excising redundant SMAS and recontouring the parotid and lower face, we addressed both functional and cosmetic issues in a single, low-morbidity procedure.

Only one other case in the literature has reported the use of a deep-plane facelift for HIV lipodystrophy, focused primarily on cervical fat excess [14]. Our case extends this concept to address midfacial atrophy and parotid fullness, highlighting the adaptability of facelift surgery to the diverse presentations of HIV-associated lipodystrophy.

As surgical strategies evolve, long-term outcomes and recurrence must be further studied. Stabilizing underlying disease processes through ART optimization and metabolic control remains essential. Nonetheless, this case demonstrates that facelift surgery can offer durable and tailored correction for a condition with few reliable alternatives.
